# Lipome intraosseux du calcanéum chez une jeune femme

**DOI:** 10.11604/pamj.2014.18.316.5035

**Published:** 2014-08-21

**Authors:** Badreeddine Alami, Siham Tizniti

**Affiliations:** 1 Service de Radiologie, CHU Hassan II, Fès, Maroc

**Keywords:** Lipome intraosseux, calcanéum, jeune femme, Intraosseous lipoma, calcaneus, young woman

## Image en medicine

Le lipome intraosseux est une tumeur bénigne rare représentant 0,1% de l'ensemble des tumeurs squelettiques. Au membre inférieur, sa localisation au calcanéum est retrouvée dans 15% des cas. Son étiopathogénie demeure imprécise et reste partagée entre origine tumorale bénigne et post-traumatique. Elle touche avec légère prédominance féminine, le sujet de la quatrième ou cinquième décade. La clinique dénuée de toute spécificité, se limite à des douleurs mécaniques de l'arrière pied; en revanche l'imagerie et particulièrement l'IRM permet de poser avec quasi-certitude le diagnostic en objectivant une tumeur de signal typiquement graisseux. Le traitement reste partagé entre l'abstention et la chirurgie avec curetage lésionnel et greffe spongieuse dans les formes douloureuses ou comportant un risque de fracture pathologique. Nous rapportons le cas d'une jeune femme, âgée de 24 ans, présentant des douleurs de type mécanique de l'arrière pied gauche, résistantes aux traitements antalgiques et chez qui une IRM a objectivé une tumeur du calcanéum se présentant en hyper signal avec liseré périphérique d'ostéosclérose en hyposignal sur les séquences T1 et T2 et perdant son signal sur la séquence de suppression de graisse, témoignant de son caractère graisseux. Nous avons retenu le diagnostic de lipome et vue que la douleur a été intense avec retentissement sur l'activité physique, la patiente a bénéficié d'un traitement chirurgical, consistant en un curetage du produit lipomateux avec greffe spongieuse.

**Figure 1:**
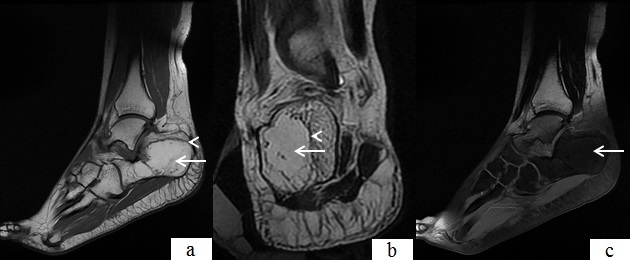
a) IRM du pied gauche, coupe sagittale en séquence T1 objectivant une lésion du calcanéum se présentant en hypersignal (flèche), bordé d'un liseré fin en hyposignal (tête de flèche); b) IRM du pied gauche, coupe coronale en séquence T2 objectivant la lésion du calcanéum en hypersignal (flèche), avec le liseré périphérique en hyposignal(tête de flèche); c) IRM du pied gauche, coupe sagittale en séquence FAT SAT objectivant un effacement du signal de la lésion du calcanéum(flèche)

